# A Survey of Regional Anesthesia Use in Greece and the Impact of a Structured Regional Anesthesia Course on Regional Techniques Knowledge and Practice

**DOI:** 10.3390/jcm10214814

**Published:** 2021-10-20

**Authors:** Kassiani Theodoraki, Eleni Moka, Alexandros Makris, Evmorfia Stavropoulou, on behalf of ESRA Hellas Working Group

**Affiliations:** 1 Department of Anesthesiology, Aretaieion University Hospital, National and Kapodistrian University of Athens, 11528 Athens, Greece; 2 Department of Anesthesiology, Creta Interclinic Hospital, 71304 Heraklion, Greece; mokaeleni@hotmail.com; 3 Department of Anesthesiology, Asklepieion Hospital of Voula, 16673 Athens, Greece; makrisalexandros@hotmail.com; 4 Department of Anesthesiology, General Hospital of Attica KAT, 14561 Athens, Greece; efistavropoulou@yahoo.gr

**Keywords:** regional anesthesia, education, regional anesthesia practice, survey, questionnaire

## Abstract

Background: Due to the growing interest in regional anesthesia (RA) techniques and the realization of the need for formalized education in them, the Greek Chapter of the European Society of Regional Anesthesia and Pain Therapy (ESRA-Hellas) has established a structured hands-on training course held annually since 2009, which is quite popular in the community of Greek anesthesiologists. The aim of the current survey was twofold: first, to provide an overview of the current practice of RA in Greece; secondly, to evaluate the effect the aforementioned training course has on participants’ knowledge and attitude towards RA. Methods: An electronic questionnaire was uploaded on SurveyMonkey and a link giving access to the questionnaire was forwarded via email to a mailing list of 825 practicing Greek anesthesiologists held in the electronic database of ESRA Hellas. The survey was totally anonymous and no identifying information was collected throughout. It contained questions relating to the anesthesiologists’ demographic characteristics, their RA practice, and information pertaining to the RA training course. Results: A total of 424 fully completed questionnaires were received, representing an overall response rate of 51.4%. Attendants of the course are more familiar than non-attendants with the performance of peripheral nerve blocks with neurostimulation and/or ultrasound guidance (*p* < 0.001). Attendants are also less likely to practice exclusively general anesthesia, more likely to use peripheral blocks for lower limb surgery, and more likely to consider taking the European Diploma of RA in comparison to non-attendants (*p* < 0.001, *p* = 0.018 and *p* = 0.002, respectively). Both cohorts consider the course of value and agree that the main reason to use regional techniques is to ensure optimal postoperative analgesia, while the main hindrance to RA practice is the lack of relevant education in the techniques, especially those under ultrasound guidance. Regarding improvement of the course, most participants suggested devoting ampler time in hands-on ultrasound practice and application. Conclusions: Greek anesthesiologists seek educational activities in the field of RA and the course seems to fulfil the majority of attendants’ expectations. There will be further effort by the organizers to improve weaknesses of the current course and undertake further educational initiatives in the field of RA according to international recommendations.

## 1. Introduction

During the past 30 years there has been a tremendous increase in the popularity of regional techniques, both for provision of anesthesia for surgery and also as part of the armamentarium of postoperative pain management. Due to the growing interest in regional anesthesia (RA) and the realization that its administration is a core skill required by all anesthesiologists, many national anesthesia societies and education committees worldwide have established training courses aiming at educating anesthesiologists in regional techniques. In light of this, the Greek chapter of the European Society of Regional Anesthesia and Pain Therapy (ESRA Hellas) has established a structured five-day intensive hands-on RA teaching module held annually since 2009. This course consists of theoretical didactic lectures on the principles of RA, the basics of ultrasound (US) machine operation, image optimization, recognition of sonoanatomy, scanning techniques, and practice on manufactured or non-human tissue phantoms (blue phantom gel models and porcine meat) as well as identification of needle insertion points and US scanning on volunteer live models. The course has so far been attended by a significant number of Greek anesthesiologists from all parts of Greece, and is popular and consistently highly ranked among participants.

In general, there is considerable variation in the practice of RA among anesthesiologists and among different countries [1,2,3,4]. In this respect, national surveys can provide valuable data for comparisons and for information concerning the transfer of evidence to clinical routine. We have previously published the results of the only nationwide survey of RA use in Greece, which aimed at investigating the trends of RA practice in the country during the year 2011 [5]. As mentioned in that publication, the collected data were considered to serve as a benchmark for future comparisons and for evaluation of the efficacy of running training programs and teaching efforts in the field of RA. That survey however was addressed to directors of Greek anesthesiology departments and not to individual anesthesiologists, therefore individual practice and routine were not clearly presented. Another limitation of the previous survey, which was in fact acknowledged in the resulting publication, was the relatively small number of responding directors, since only 66 completed questionnaires were collected out of the 230 anesthesiology departments nationwide.

The aim of the current online survey was twofold: first, to conduct a nationwide voluntary anonymous response survey to document the current practice of RA in Greece. The difference from the previous survey lay in the fact that we aimed at individual anesthesiologists in the hope that responses would be more representative of individual practices rather than departmental ones and that the survey would provide a comprehensive overview of the current status of individual RA practice. Secondly, we sought to elucidate whether the aforementioned training course in RA, which is now over its 10-year anniversary, has achieved its goals in offering targeted education and consolidating knowledge and skills in the field of RA amongst Greek anesthesiologists. Our ultimate goal was to elaborate on the course’s strengths and weaknesses and to identify deficiencies with the target of paving the way for instructor feedback, self-assessment, and improvement, thus acting as an internal quality improvement project.

## 2. Methods

### 2.1. Study Design and Questionnaire

The survey was endorsed by the Scientific Committee of ESRA Hellas and was undertaken from 26 February 2020 to 26 March 2020. All methods were performed in accordance with relevant guidelines and regulations and followed set research practices for surveys [6]. After obtaining approval from the Institutional Review Board of the Aretaieion University Hospital of the National and Kapodistrian University of Athens (194/25-02-2020), an electronic questionnaire was compiled and uploaded on SurveyMonkey (SurveyMonkey Inc., San Mateo, CA, USA, www.surveymonkey.com (accessed on 30 April 2021)), a web-based, commercially available survey research platform tool that allows users to create their own surveys, using question format templates. The questionnaire had been thoroughly reviewed for content and structure by two senior certified consultants with vast experience in RA to ensure validity and clarity and to correct ambiguities and misinterpretations. It was agreed upon by consensus between them. It consisted of questions focusing on anesthesiologists’ demographic characteristics, their RA practice as well as information relating to the aforementioned RA course. The questionnaire was tested in different browsers and platforms to ensure smooth conduct. It was further validated by pilot testing on five senior anesthesiologists who provided feedback that was incorporated into the final survey. On average the sample questionnaire took eight minutes to complete. A secure link giving access to the online questionnaire created using the SurveyMonkey website was sent via email to a mailing list of 825 practicing Greek anesthesiologists that was provided to the major officers of the society by an administrative authority in the Ministry of Health. All anesthesiologists working in state, private, and academic hospitals according to the data of this list were invited to participate since our objective was to reach a representative sample nationwide. The questionnaire was accompanied by a short narrative explaining the purpose of the survey and inviting all colleagues to participate; however, no monetary compensation or any other incentive was provided for completion of the questionnaire. The survey was open for one month and the respondents could choose either desktop or mobile device to complete it via the secure link; however, each IP address was allowed to complete the survey once. Two subsequent reminders were sent thereafter via follow-up emails and through popular electronic social media (i.e., the Facebook page of ESRA Hellas) within the period of the month during which the survey was open, kindly requesting anesthesiologists to complete the questionnaire. After initial mailing and the subsequent follow-up reminders, the survey was closed one month after the initial distribution. All responses were collected at the end of the month through the SurveyMonkey website. The survey was totally anonymous and no identifying information was collected. The study was registered post hoc at the clinicaltrials.gov website (ClinicalTrials.gov ID: NCT04406025).

The questionnaire contained 32 multiple-choice questions (MCQs) and one free-text question. At the end of the questionnaire, informed consent was obtained from respondents to the survey to use their anonymized data for research purposes (File S1). In the MCQs, the selection of the best answer was only possible and rating scales were included in some of the questions, depending on the context. The survey consisted of two parts: firstly, it included basic demographic information about the anesthesiologists’ hospital job position and level, years of clinical practice, type of hospital, techniques of anesthesia used in the hospital, percentage of operations performed under RA in the respective hospitals, type of equipment available in the hospital allowing the performance of regional anesthetic techniques and personal preference, and the frequency and practice of the physicians regarding RA techniques in general in the context of their current routine practice. Participants were additionally asked about their knowledge of and confidence in performing neuraxial and peripheral nerve blocks (PNBs), the utilization of catheters after neuraxial or PNBs for provision of postoperative analgesia, and the use of US and/or nerve stimulation guidance in their practice of regional techniques. There were also questions pertaining to respondents’ personal beliefs on the advantages of RA and on factors hindering its broad application in the participants’ working environments.

The second part of the questionnaire focused on the experience of the participants with the aforementioned RA course. Those that had attended the course were asked about the main reason motivating them to participate in it and whether they felt that the course added to their knowledge, confidence, and competence regarding neuraxial and peripheral RA techniques. Information was sought about whether, after the course, their motivation to engage in RA increased, whether they asked for the provision of relevant equipment in their working environments, and whether they were met with any obstacles in this provision. They were also asked about whether they considered the course of value in RA education, about whether they would suggest attending it to other colleagues and were asked to suggest areas for improvement for the course. Finally, those anesthesiologists who had not attended the course were asked about their reason for not doing so and, irrespective of their lack of attendance, whether they thought that the course was valuable in RA education. Finally, the survey was concluded with a general question addressed to all participants regarding their thoughts about participation in the examination for the European Diploma of Regional Anesthesia (EDRA).

### 2.2. Data Analysis

Data were coded and were stored anonymously on the SurveyMonkey host server. They were then downloaded for analysis into an Excel Database and were subsequently analyzed with the SigmaPlot for Windows v.13.0 statistical software (Systat Software, Inc., San Jose, CA, USA). Descriptive statistics (frequency distributions) were computed to summarize the data. Differences in characteristics and attitudes between anesthesiologists who had exposure to the RA course and those who did not were analyzed with chi square analyses, with Yate’s correction and Fisher’s exact test, as appropriate. For the level of knowledge in central and peripheral blocks and in order to allow comparisons, a 5-point Likert scale was used and categories were coded as follows: 0, no knowledge; 1, little knowledge; 2, average knowledge; 3, very good knowledge; 4, expertise. For the comparison in the levels of knowledge between the anesthesiologists who attended the course and those who did not, the Mann–Whitney U-test for independent samples was used while for the comparison in the levels of knowledge in the performance of blocks among all respondents, the Wilcoxon signed-rank test was used. Results are presented as number (frequency) for categorical variables and as median (25th–75th percentile) for the results of the Likert scale and were considered significant at the *p* < 0.05 level.

## 3. Results

Τhe survey was forwarded to 825 practicing anesthesiologists. A total of 424 responses were obtained with fully completed questionnaires, corresponding to an overall response rate of 51.4%. Responders to the survey were a mix of attendants (*n* = 189) and non-attendants (*n* = 235) of the regional anesthesia course. The majority of the respondents (33.7%) reported that they work in central National Health Service (NHS) hospitals and that they are consultants with more than 10 years of experience in anesthesia practice (42.7%). Most people (40.3%) stated that they work in hospitals where all types of anesthetic techniques are practiced (general anesthesia, neuraxial blocks, and peripheral blocks with both neurostimulation and US guidance) and most (30.6%) also reported that the percentage of operations under RA in their practicing hospitals is between 31–50%. Most respondents also ascertained that they practice in hospitals where they have ready access to both neurostimulators and a US machine and that a local anesthetic systemic toxicity (LAST) kit is available in their departments (57.0% and 69.8% respectively).

The vast majority of respondents (94.8%) stated that they know about the RA course organized annually by ESRA Hellas but more than half of them (55.4%) have not attended it. The majority of anesthesiologists who have attended the course (89.4%) stated that their main motive for doing so was to improve knowledge and skills in RA. Most anesthesiologists who have attended the course agreed that it contributed considerably to acquiring knowledge in central neuraxial blocks, in central neuraxial blocks under US guidance, in PNBs with neurostimulation, and in PNBs under US guidance (with percentages of 58.7%, 40.7%, 46.0%, and 49.7%, respectively). However, when asked whether the knowledge they acquired changed their everyday practice of RA, the majority of them (46.0%) stated it changed their everyday practice only a little, attributing this fact to the ongoing lack of competence in US use. Nonetheless, the majority of anesthesiologists attending the course (59.2%) declared that it provided the motive for requesting the provision of relevant equipment from their hospital services.

When asked about their personal anesthesia practice, most respondents (39.8%) stated that this mainly consists of general anesthesia and neuraxial blocks without US guidance. However, those who have not attended the course are more likely to practice mainly general anesthesia as compared to those who have attended the course (*p* < 0.001). In contrast, anesthesiologists who have attended the course are more familiar with providing all anesthesia techniques (general anesthesia, central blocks, and PNBs with neurostimulation and/or US guidance) in comparison to non-attendant counterparts (*p* = 0.008). In addition, when specifically asked regarding personal practice in PNBs, the majority of respondents (31.1%) stated that they seldom try to perform them because of a lack of confidence and expertise. However, when responses to this question were analyzed in terms of attending the workshop, anesthesiologists who had attended the workshop were twice as likely to perform PNBs under US guidance compared to those who had not attended it (*p* < 0.001). Additionally, there was a higher chance that anesthesiologists who had not attended the course work in hospitals where operations amenable to PNBs (such as orthopedic cases) do not take place, as compared to those who have attended the RA course (*p* = 0.006). Most respondents (33.2%) also stated that their primary choice for anesthesia technique in lower limb surgery is a neuraxial block. Nonetheless, there was a higher chance of performing a PNB in lower limb surgery among those who had attended the workshop (*p* = 0.018) and a higher chance of working in a hospital where such surgery does not take place among those who had not attended the workshop (*p* = 0.033), (Table 1).

**Figure 1:**
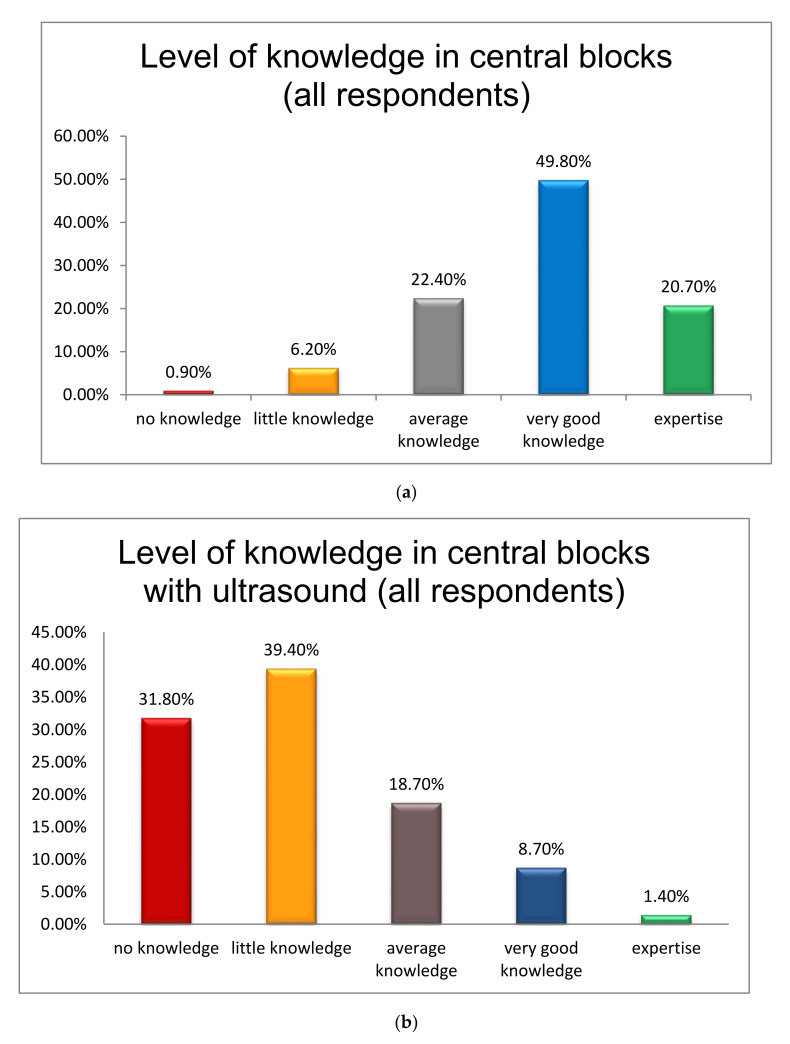

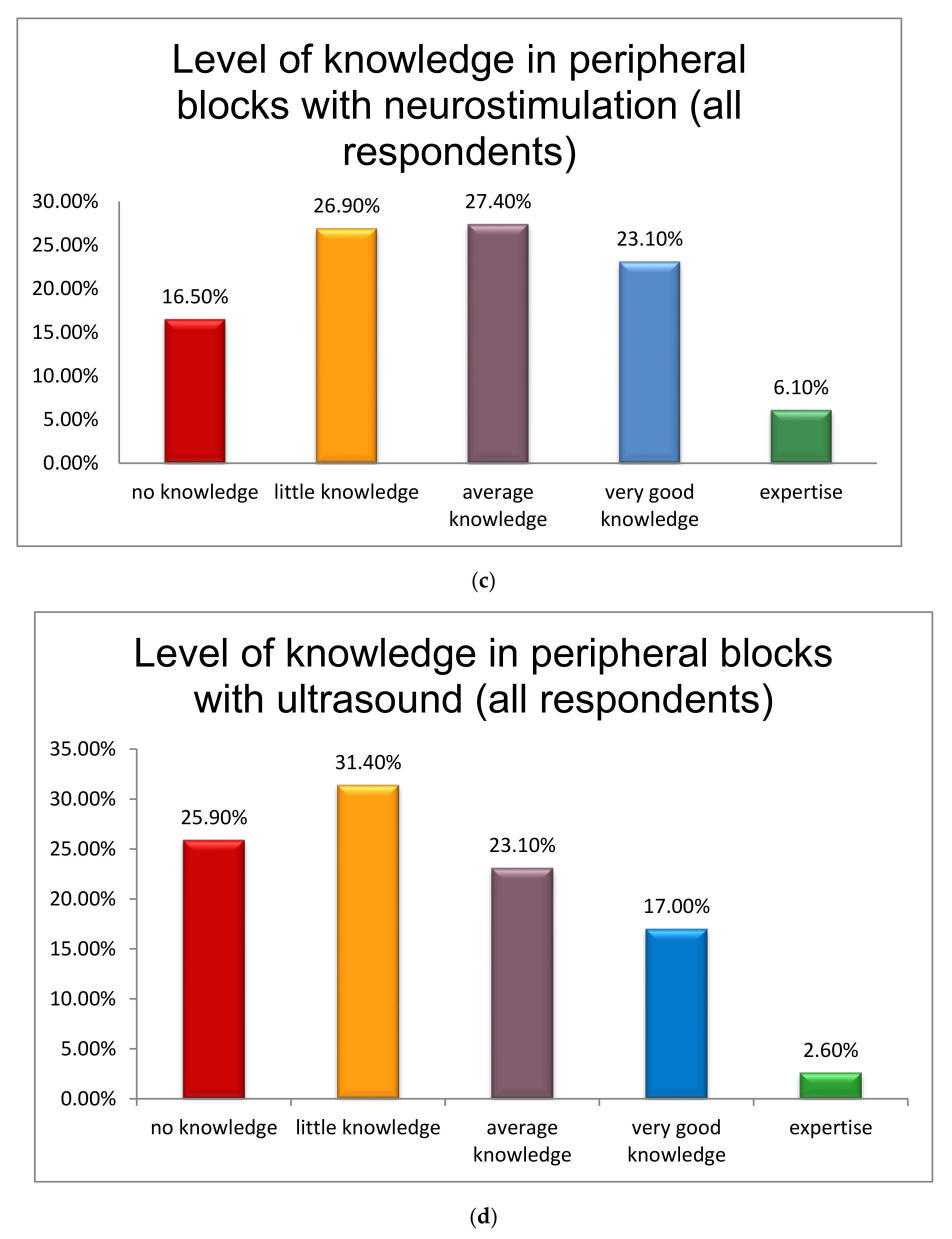
(**a**) Level of knowledge in central blocks (all anesthesiologists who responded to the survey) (**b**) Level of knowledge in central blocks with ultrasound guidance (all anesthesiologists who responded to the survey) (**c**) Level of knowledge in peripheral blocks with neurostimulation (all anesthesiologists who responded to the survey) (**d**) Level of knowledge in peripheral blocks with ultrasound guidance (all anesthesiologists who responded to the survey).

Respondents were also asked regarding their level of knowledge in central and peripheral blocks. The majority of anesthetists (49.8%) reported very good knowledge in central blocks, with no difference between those who had and those who had not attended the course (3 (3–3) vs. 3 (2–3), *p* = 0.059) (Figure 1a). However, most of the respondents (39.4%) declared that they have little knowledge in the performance of central blocks under US guidance, with a significant difference between those who had and those who had not attended the course (1 (1–2) vs. 1 (0–1), *p* < 0.001) (Figure 1b). As to PNBs, most respondents (27.4%) noted their average knowledge in the performance of these blocks aided by neurostimulation with the difference between those who had and those who had not attended the course also being significant (2 (1–3) vs. 1 (1–2), *p* < 0.001) (Figure 1c). Lastly, when asked about their knowledge in the performance of PNBs under US guidance, most respondents (31.4%) reported that they had little knowledge in doing so, with a significant difference continuing between those who had and those who had not attended the course (2 (1–3) vs. 1 (0–2), *p* < 0.001) (Figure 1d).

According to the results of the survey, knowledge in PNBs with neurostimulation among all respondents far exceeds their knowledge in PNBs with US guidance (2 (1–3) vs. 1 (0–2), *p* < 0.001). Similarly, respondents are more familiar with the performance of PNBs with US as compared to the performance of central blocks with US (1 (0–2) vs. 1 (0–2), *p* < 0.001). Lastly, the difference in the performance of central blocks with traditional anatomic landmarks as compared to the performance of these blocks via US guidance is also highly significant (3 (2–3) vs. 1 (0–2), *p* < 0.001).

Regarding the use of catheters, the majority of respondents (72.4%) often use an epidural catheter for the provision of postoperative analgesia, with no difference between those who have and those who have not attended the course (*p* = 0.176). However, when asked about the use of catheters after PNBs, a high percentage of respondents (41.5%) stated that they never use a catheter for the provision of postoperative analgesia. However, the higher use of catheters among those who attended the course was demonstrated and the difference with non-attendants was highly significant (*p* < 0.001), (Table 1).

Both groups agreed that the main reason for performing RA techniques is the provision of more optimal postoperative analgesia with no difference between attendants and non-attendants (*p* = 0.573). Other reasons quoted were safety, improved outcomes, the reduction of complications, and the reduced cost of hospitalization, in descending order. The primary impediment to the wider application of regional techniques is the lack of education in them, with no difference between those who attended and those that did not attend the course (*p* = 0.991). Other key barriers listed were the long duration of RA procedures, reluctant surgeons, patient refusal, concern for potential complications, and fear of failure, in descending order (Table 1).

**Figure 2:**
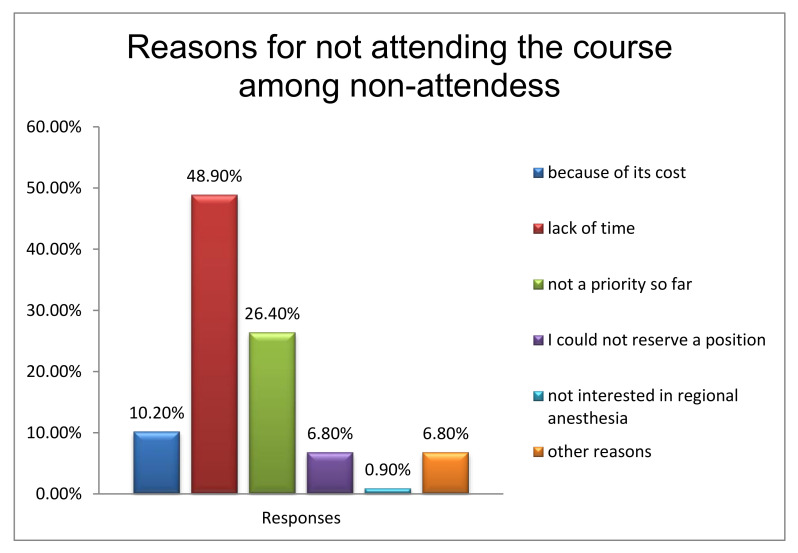
Reasons for not attending the regional anesthesia course among anesthesiologists who had not attended the course.

**Figure 3:**
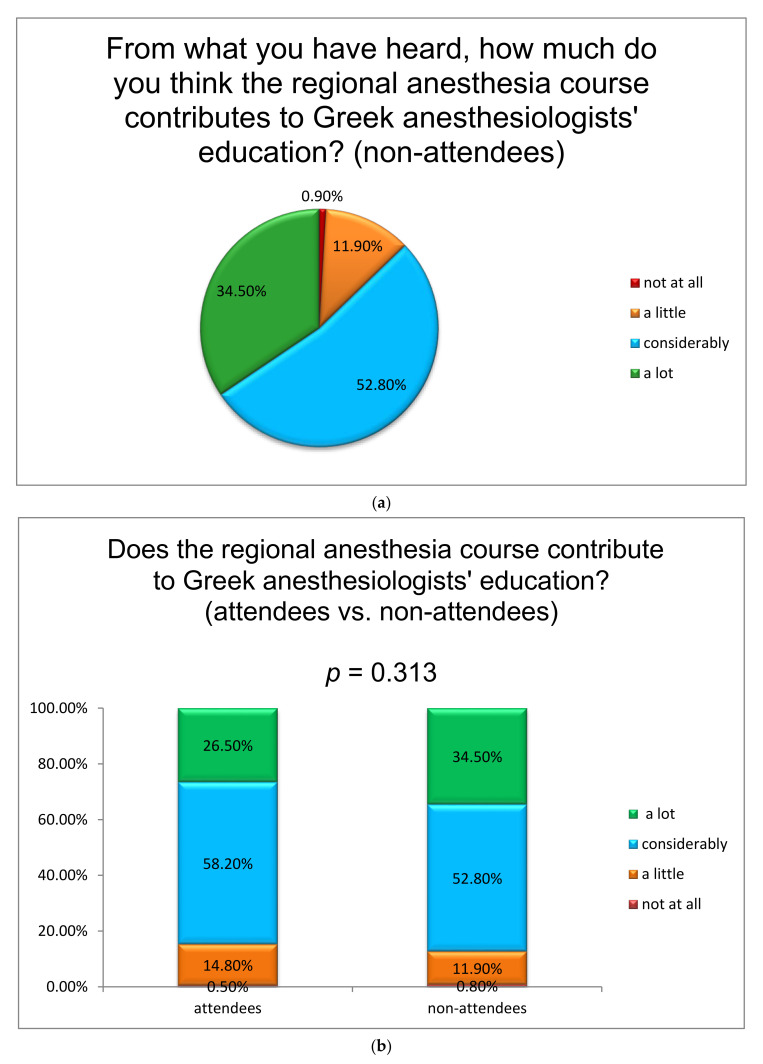
(**a**) Contribution of the course to regional anesthesia education of Greek anesthesiologists according to people who have not attended the regional anesthesia course; (**b**) Contribution of the course to regional anesthesia education of Greek anesthesiologists (attendants versus non-attendants of the regional anesthesia course).

A high percentage of the anesthesiologists (88.4%) who attended the RA course would recommend its attendance to their colleagues, while the main reason for non-attendance among non-attendees is their busy work schedule (48.9%) (Figure 2). However, both groups agree that the course contributes considerably to Greek anesthesiologists’ education in RA (Figure 3a), with no difference between those who did and those who did not attend the course (*p* = 0.313) (Figure 3b).

**Figure 4:**
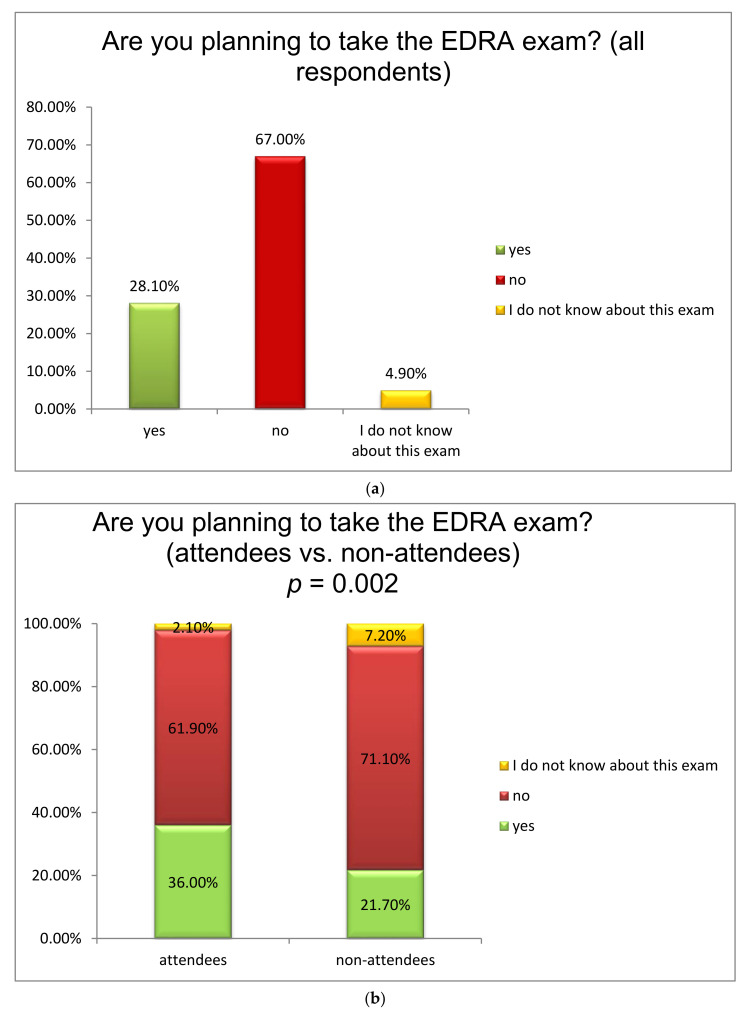
(**a**) Willingness to take the European Diploma of Regional Anesthesia (EDRA) exam (all anesthesiologists who responded to the survey); (**b**) Willingness to take the European Diploma of Regional Anesthesia (EDRA) exam (attendants versus non-attendants of the regional anesthesia course).

Finally, although the majority of respondents to the survey (67.0%) had not or did not intend to take the EDRA exam (Figure 4a), those who attended the course were more likely to consider taking the exam as compared to those who did not attend the course (*p* = 0.002) (Figure 4b).

## 4. Discussion

The main findings of this survey were that anesthesiologists who have attended the RA course are more knowledgeable regarding the performance of peripheral blocks with neurostimulation and/or US guidance as compared to those who have not attended the course. Attendees are also less likely to practice exclusively general anesthesia in their hospitals, more likely to attempt RA techniques and the insertion of peripheral nerve catheters, and more likely to consider taking the EDRA exam as opposed to non-attendees. Both cohorts consider the course of value and agree that the main reason for applying RA techniques is to ensure superior postoperative analgesia and that the main barrier in RA practice is the lack of relevant education in the techniques.

Our study also highlighted some interesting findings regarding the RA pattern of practice. The majority of respondents declared that their technique of choice for lower extremity surgery is a neuraxial block as opposed to a peripheral block, with no difference between attendants and non-attendants of the course. The ease of use, fast learning curve, and high success rate of neuraxial blockade, and the broad exposure of residents to obstetric anesthesia, with its preponderance of neuraxial approaches, may underlie this finding [7,8,9,10,11,12,13,14,15]. In a study evaluating confidence levels, residents at the end of their training did not report confidence in performing PNBs, to which they have little exposure [9]. Confidence is a substantial factor in one’s ability to continue to perform blocks beyond residency and if graduating residents feel inadequately prepared for a variety of regional techniques, they will hardly use techniques in which they lack expertise [16,17,18,19]. In other words, lost training opportunities during residency can lead to graduates failing to incorporate techniques into future practice [20]. Consequently, infrequent use at consultant level makes attaining and retaining proficiency difficult; therefore, it is of paramount importance that education in RA is continuous post-residency, so that the pool of experienced teachers increases and stays abreast of the latest advances. Another aspect of the same problem is the fact that traditionally and before the era of US, PNBs for the lower extremities were practiced less frequently and were considered technically more demanding and cumbersome because of the need to perform multiple blocks to anesthetize the entire limb, whereas this was not the case for peripheral blocks of the upper extremity. This trend has been reported in various surveys [1,3,10,21,22]. Additionally, neuraxial anesthesia, in which anesthesiologists feel more confident as mentioned above, is usually a viable alternative for lower extremity surgery, whereas there is no alternative for upper extremity blocks. It appears according to more recent surveys as though lower extremity PNBs are gaining ground over upper extremity techniques and have now an upgraded role in clinical practice [23,24]. At present, we are witnessing an increasing trend of PNB use over neuraxial blocks especially in orthopedics and the focus of training has shifted accordingly [24,25,26]. The advent and more universal application of US, which offers the ability to visualize neural structures in relation to surrounding tissues, needle advancement in real time, and local anesthetic spread around nerves, as well as today’s emphasis on ambulatory surgery and “fast-tracking” of patients, might account for this tendency of equilibration [27,28]. Although the enhanced popularity of PNBs in recent years has been substantiated in other regions, it appears that this is not the case in Greece as yet. According to the results of our survey, it seems that more needs to be done to fill the gap in confidence related to PNBs, to remedy training deficits, and to shift the focus of RA education from neuraxial to PNBs in accordance to international trends and recommendations for a diverse case mix in training programs.

The popularity of central nerve blocks was confirmed in this survey and is in accordance with the results of the previous survey performed in the Greek region [5] since the majority of anesthesiologists who responded to the survey ascertained their very good level of knowledge in central blocks, which did not seem to be affected by attendance of the course. Greek anesthesiologists also often use epidural catheters for the provision of postoperative analgesia, whereas this practice is not affected by the attendance or not of the course. This is in accordance with an older survey highlighting the popularity of epidural catheter use in Greek anesthesia departments [29]. Nevertheless, this is not the case regarding the use of peripheral nerve catheters, since a high percentage of respondents do not use this form of postoperative analgesia. It appears however that the attendance of the course has an impact on the use of peripheral nerve catheters as there are significant differences in the use of peripheral catheters between attendants and non-attendants.

US-guided regional techniques have enhanced our ability to achieve effective and consistent blocks, and the implementation of US guidance has been hailed as the new gold standard as far as efficacy and safety are concerned [30]. The main barriers to US use, both at an institutional and personal level, are unavailability of equipment and lack of training [30,31,32]. The cost-effectiveness of US-guided regional nerve blocks in comparison to landmark techniques has also been noted [33]. However, respondents to our survey overall confirmed that their knowledge of US application in peripheral block performance is inferior to using neurostimulation guidance in the performance of such blocks and that their knowledge and use of US in central block performance is severely inferior to the performance of central blocks with anatomical landmarks only. It is common knowledge that US-focused workshops play a vital role in the acquisition of the necessary skills to both safely and effectively practice RA techniques under US guidance [34]. The gradual development of factual knowledge and motor skills is essential for residents and anesthesiologists in post-residency posts alike, while the integration of multiple technical and cognitive skills is necessary to achieve proficiency in the long term [19]. The Greek RA course seems to fulfil some of these goals since, according to the results of this survey, attendees of the RA workshop ascertained that the workshop contributed significantly to the acquisition of the theoretical knowledge in all aspects of RA, including the basics of US guidance for nerve localization. Therefore, the course seems to fulfil (at least partially) the knowledge gap of training opportunities in the Greek region.

However, the majority of participants in the course, by stating that the course changed their practice only a little, seem to be reluctant to universally incorporate US use in their everyday routine, admitting that even after the course, they lack the confidence in broad US application and implementation in their daily practice. It appears therefore that despite the intensive structure of the Greek RA workshop and an attempt from the instructors to teach the basics of the aforementioned three components, participants feel that more is needed in terms of quantity of learning so that key competencies taught can safely be extrapolated to the clinical realm and true day-to-day incorporation of RA in routine practice can be achieved [35]. In a study by Barrington and colleagues, in which the authors examined the amount of training required for naïve learners to identify the necessary anatomy for ultrasound-guided axillary block, they deduced that sonographic competence was achieved after eight to ten practice sessions [36]. It appears therefore that more teaching time, including frequent exposure to learning opportunities and learning aids that help shorten a trainee’s learning curve (peer-to-peer learning, participation in e-learning modules, and hands-on workshops), are required to bring novices from baseline to competence and to enable them to effectuate a change in practice [21,37,38]. It has also been shown that repetitive opportunities are essential to reinforce learning and enable the acquisition of procedural skills [39]. This was actually reflected in participants’ replies when asked to provide written feedback and make suggestions towards the improvement of the course in the only free-text question of the survey, where many people suggested expanding time allocated in US hands-on practice and application. This might be of particular importance in the case of US use for the performance of the central blocks as, according to our survey, the adoption of US as an aid in the performance of central blocks considerably lags behind US use for peripheral blocks, and most respondents never or seldom use it for neuraxial anesthesia. This discrepancy has been highlighted in other studies and efforts towards its reversal could provide a valuable tool in the anesthesiologists’ armamentarium when facing patients with challenging anatomy and generally in cases where one might consider aborting the effort for regional anesthesia [18,32,40]. In fact, with more recent technological advances, the use of US has been expanded to include guiding more technically demanding procedures, such as neuraxial blockade.

Still, it appears that the course, despite its weaknesses, has created the foundation for the consolidation of basic knowledge in the performance of central and peripheral blocks via US guidance. Participants had statistically significant gains in knowledge as compared to non-attendees. Additionally, despite the aforementioned difficulties, it seems that the course fulfils the target of familiarizing participants with RA practice, by creating interest and motivation in the use of RA procedures and perhaps enabling long-term retention of the skills taught. In fact, as it was shown in our survey, anesthesiologists who have attended the workshop are less likely to administer exclusively general anesthesia and more likely to use all types of anesthetic techniques and in particular peripheral blocks for lower limb surgery as opposed to those who have not attended the course, and these differences were statistically significant. Training can act as a major driving factor in the application of RA [9]. It appears therefore that the course provides participants with the opportunity to acquire new skills, to develop a larger repertoire of techniques, and to return to their clinical settings with new knowledge and strategies and thus to be more conducive to expanding their practice.

Teaching has been shown to dramatically increase the number of blocks performed and anesthesiologists have realized that didactic teaching can supply them with a basic framework of factual knowledge [17,37]. This is reflected in our survey since the vast majority of people who attended the course (almost 90%) attested that they did so in order to improve knowledge and skills in RA. Another noteworthy finding of the study is that all respondents to the survey consider that the RA course contributes to Greek anesthesiologists’ education with no difference between attendants and non-attendants. It seems that the course is quite popular and people who have not attended it also rate it highly and attribute non-attendance to their busy work schedule and lack of time. It appears that anesthesiologists’ attitude has changed in recent years towards seeking high quality education activities, therefore they highly value opportunities for structured hands-on training.

The general view of respondents regarding reasons for performing regional techniques is in accordance with other studies. In fact, the notion that the main reason to use RA is to provide optimal postoperative analgesia has barely changed throughout the years [41]. On the other hand, respondents identified the lack of training as the major hindrance for broader RA application in everyday practice, a fact that has been extensively pinpointed previously [1,42]. It is of interest that in an older study performed in the UK, the main barrier to RA was the length of time required to establish the block [41]. It appears that the realization of today’s training opportunities along with the considerable assistance of novel techniques aiding in the performance of regional blocks and in the reduction of performance time account for this change in mindset. In our survey, surgeon- and time-related reasons took second and third place as key barriers in the performance of RA. Interestingly, the percentage corresponding to reluctance from surgeons is higher than the one corresponding to reluctance from patients. Lack of support from surgeons and erroneous perceptions are ongoing institutional challenges. A survey of Canadian orthopedic surgeons revealed that only 40% of them directed their patients towards RA [43]. Surgeons not in favor are most probably unfamiliar with the benefits of PNBs and think that RA is a complex procedure, which results in delays and unpredictable success rates with possible conversion to general anesthesia. In such situations, it has been shown that the availability of designated block rooms can expedite operating room flow, provide an unhurried and less stressful environment for teaching, reduce delays between cases, and overcome logistical impediments in fast-paced clinical environments and high-volume institutions, where rapid turnover of cases is of the essence [44,45]. As to the availability of Intralipid, which is crucial in the management of LAST, the fact that Intralipid was stored in only 70% of hospitals according to the replies provided shows that more needs to be done to create awareness about LAST and to conform to current recommendations that a lipid rescue kit should be available in any setting in which RA is practiced [46].

Finally, it is perhaps unsurprising that anesthesiologists who have attended the course have a greater intention to attempt taking the EDRA diploma in comparison to those who have not. This is in accordance with the fact that anesthesiologists who attended the course are less likely to administer solely general anesthesia and more likely to work in orthopedic hospitals where a variety of regional techniques are attempted. Anesthesiologists who have attended the course seem to be more interested in acquiring an extra accreditation related to regional anesthesia career-wise and in proving that their knowledge encompasses the field of RA, since proof of RA expertise may have employment implications [38]. Therefore, the willingness to take the exam may be an indirect indication of a greater motivation for an RA qualification.

### Strengths and Limitations

A limitation of the current survey, as with other surveys of this kind, is the non-responder bias. There is always a risk of bias caused by clinicians left out of the survey, as anesthesiologists with little interest in RA might have not shown interest in completing a questionnaire forwarded by an RA Society. Although we took measures to optimize the response rate, we do not know whether non-respondents would have answered in the same manner as respondents. Another limitation is the fact that expertise or lack thereof was based on self-estimation and therefore participants’ perception of competency and of procedural performance was subjective. The objective estimation of expertise cannot be based on a survey tool but rather requires systemic theoretical and practical evaluation of respondents, which however was beyond the scope of the current study. An additional limitation is the fact that the questionnaire used was not validated or constructed by an expert in the design of surveys; however, it was subjected to thorough revision and pilot testing before final distribution. Finally, our survey was a cross-sectional study, not reflecting the longitudinal changes in training. However, our survey had many strengths. Firstly, we achieved a satisfactory response rate (over 50% of the targeted individuals), within the range of previously published similar surveys [1,4,10,14]. Therefore, we consider we provided useful insights into the nationwide practice of RA and the evaluation of the RA hands-on workshop. It has been recommended that the minimal number of survey responses required for survey validity is ten times the number of questions [47]. The current 33-question survey required at least 330 responses and we received 424, which is a reasonable response rate. Notably, the anonymous design chosen reduces reservations in responding and the tendency to provide idealized answers, resulting in self-report bias. Additionally, questionnaires without a lot of open questions maximize response rate. Secondly, we consider that we obtained representative information about the practice and opinions in the whole country, since we had responses from a variety of health institutions from across all regions of Greece, including academic and non-academic centers, community hospitals, and private practice settings. Therefore, this preliminary exploratory survey could create the basis for future comparisons and could be an important step towards future European or international initiatives using validated questionnaires to assess the impact of other educational activities in the field of RA with a wider geographical scope.

## 5. Conclusions

The results of the current survey highlight that despite its weaknesses, a dedicated RA course may increase subsequent RA practice by concentrating learning experience into a focused period. At a national level, future advances in RA will be highly dependent on the quality of education. In the previous survey about RA practice in Greece, the lack of a formal stepwise program incorporated in the curriculum of residency had been emphasized as a significant shortcoming to systematic training in RA techniques [5]. In the last couple of years, the situation has changed as relevant administrative authorities have realized the importance of standardized training and formalized teaching programs in many medical specialties including anesthesiology. Training programs have started moving away from apprenticeship models, which prevailed in the last several decades and provided inconsistent learning experiences, towards competency-based methods of education. Thus, the curriculum of the specialty has been redesigned, amendments have been suggested, deficiencies have been identified and a structured program of specific rotations offering more targeted education in RA by incorporating formal RA rotations has now been officially integrated into the residency curriculum of Greek anesthesiologists. The aim is to make RA techniques an integral part of professional training during residency and to ensure trainees receive exposure to both conceptual knowledge and practical experience, which can significantly impact the utilization of the techniques post-residency. Hopefully this fact, along with the improvement of the present course as well as future educational initiatives specifically targeting PNBs, the follow-up of innovations, and especially the increased exposure to US teaching might greatly enhance the training process in RA by not only providing core skills but also by creating the basis for the implementation of a solid curriculum in RA training.

## Figures and Tables

**Table 1:** Personal practice of respondents in aspects of regional anesthesia techniques; results are presented as number (percentage %); *p* value for the comparison between students who attended and did not attend the course; bold numerals denote a significant difference between people who attended the regional anesthesia course and those who did not (*p* < 0.05).

	All Respondents (*n* = 424)	People Who Attended the Regional Anesthesia Course (*n* = 189)	People Who Did Not Attend the Regional Anesthesia Course (*n* = 235)	*p*-Value
Personal practice in anesthesia provision				
- mainly general anesthesia	49 (11.5)	10 (5.3)	39 (16.6)	**<0.001**
- general anesthesia and central blocks	169 (39.8)	75 (39.7)	94 (40.0)	0.973
- general anesthesia, central blocks, and peripheral, blocks with neurostimulation	63 (14.8)	27 (14.3)	36 (15.3)	0.873
- general anesthesia, central blocks, and peripheral blocks with both neurostimulation and ultrasound	144 (33.9)	77 (40.7)	66 (28.1)	**0.008**
Personal practice in peripheral blocks				
- use of ultrasound (confidence and expertise)	128 (30.2)	79 (41.8)	49 (20.9)	**<0.001**
- use of neurostimulation (lack of confidence and expertise in ultrasound)	66 (15.6)	26 (13.8)	40 (17.0)	0.431
- use of neurostimulation (no ultrasound machine available)	35 (8.3)	15 (7.9)	20 (8.5)	0.971
- use of anatomic landmarks only	4 (0.9)	1 (0.5)	3 (1.3)	0.775
- no peripheral blocks (no confidence, do not know the technique)	132 (31.1)	52 (27.5)	80 (34.0)	0.181
- no peripheral blocks (no suitable operations in my hospital)	59 (13.9)	16 (8.5)	43 (18.3)	**0.006**
Personal practice in lower limb surgery				
- neuraxial block	141 (33.2)	63 (33.3)	78 (33.2)	0.942
- peripheral block	133 (31.4)	71 (37.6)	62 (26.4)	**0.018**
- general anesthesia	84 (19.8)	34 (18.0)	50 (21.3)	0.471
- no lower limb surgery in my hospital	66 (15.6)	21 (11.1)	45 (19.1)	**0.033**
Epidural catheters				
- often	307 (72.4)	138 (73.0)	169 (71.9)	0.176
- occasionally	90 (21.2)	42 (22.2)	48 (20.4)	
- never	11 (2.6)	6 (3.2)	5 (2.1)	
- I do not do epidurals	16 (3.8)	3 (1.6)	13 (5.6)	
Catheters in peripheral nerve blocks				
- never	176 (41.5)	68 (36.0)	108 (46.0)	<0.001
- yes, for 24 h	25 (5.9)	23 (12.1)	2 (0.8)	
- yes, for 2–3 days	32 (7.6)	30 (15.9)	2 (0.8)	
- I do not perform peripheral blocks	191 (45.0)	68 (36.0)	123 (52.4)	
Main reasons for performing regional anesthesia				
- regional anesthesia is safe	79 (18.6)	34 (18.0)	45 (19.1)	0.573
- regional anesthesia improves outcome	48 (11.3)	25 (13.2)	23 (9.8)	
- regional anesthesia decreases cost of hospitalization	17 (4.0)	5 (2.6)	12 (5.1)	
- regional anesthesia decreases the incidence of complications	42 (9.9)	19 (10.1)	23 (9.8)	
- regional anesthesia ensures superior postoperative analgesia	208 (49.1)	90 (47.6)	118 (50.2)	
- all of the above	30 (7.1)	16 (8.5)	14 (6.0)	
Obstacles in performing regional anesthesia				
- lack of education	287 (67.7)	127 (67.2)	160 (68.1)	0.991
- time-consuming	62 (14.6)	29 (15.3)	33 (14.0)	
- patients negative	22 (5.2)	9 (4.8)	13 (5.5)	
- surgeons negative	45 (10.6)	21 (11.1)	24 (10.2)	
- high percentage of lack of success	3 (0.7)	1 (0.5)	2 (0.9)	
- significant percentage of complications	5 (1.2)	2 (1.1)	3 (1.3)	

## Data Availability

The datasets generated during and/or analyzed during the current study are available from the corresponding author on reasonable request.

## Supplementary Materials

The following are available online at https://www.mdpi.com/article/10.3390/jcm10214814/s1, File S1: Survey.

## Abbreviations

The following abbreviations are used in this manuscript:

ESRA	European Society of Regional Anesthesia and Pain Therapy
ESRA Hellas	Greek Chapter of the European Society of Regional Anesthesia and Pain Therapy
US	Ultrasound
PNBs	Peripheral Nerve Blocks
NHS	National Health Service
LAST	Local Anesthetic Systemic Toxicity
