# Supplementary material for: A Survey of Regional Anesthesia Use in Greece and the Impact of a Structured Regional Anesthesia Course on Regional Techniques Knowledge and Practice
Source: J Clin Med. 2021 Oct 20;10(21):4814. doi: 10.3390/jcm10214814 (PMC8584817; doi:10.3390/jcm10214814)
Supplement: Supplementary file 1 [file jcm-10-04814-s001.zip › jcm-1388917-supplementary.pdf]

File S1. Survey

Part I. Regional Anesthesia Practice

1. You are a:

-resident/trainee □

-consultant with < 5 years of experience □

-consultant with 5–10 years of experience □

-consultant with >10 years of experience □

2. You work in a:

-central NHS Hospital □

-district NHS Hospital □

-central University Hospital □

-district University Hospital □

-military Hospital □

-central private Hospital □

-district private Hospital □

-other institutions □

3. In the Hospital you work:

-mainly general anesthesia is performed □

-general anesthesia and neuraxial blocks are performed □

-general anesthesia, neuraxial blocks and peripheral blocks with neurostimulation are performed □

- general anesthesia, neuraxial blocks and peripheral blocks with both neurostimulation and ultrasound are performed □

4. The percentage of operations under regional anesthesia in your Hospital is:

-<10% □

-10–20% □

-20–30% □

-31–50% □

->50% □

5. The available equipment on your Hospital for the performance of regional anesthesia techniques is:

-neurostimulator □

-ultrasound machine □

-both neurostimulator and ultrasound machine □

-none □

6. Is there a local anesthetic toxicity kit in your Hospital?

-yes □

-no □

- I don't know what this is □

7. What is your personal practice in anesthesia provision?

-mainly general anesthesia □

-general anesthesia and central blocks □

-general anesthesia, central blocks and peripheral, blocks with neurostimulation □

-general anesthesia, central blocks and peripheral blocks with both neurostimulation and ultrasound □

8. What is your personal practice in peripheral blocks?

-use of ultrasound (confidence and expertise) □

-use of neurostimulation (lack of confidence and expertise in ultrasound) □

-use of neurostimulation (no ultrasound machine available) □

-use of anatomic landmarks only □

-no peripheral blocks (no confidence, do not know the technique) □

-no peripheral blocks (no suitable operations in my Hospital) □

9. What is your personal practice in lower limb surgery?

-neuraxial block □

-peripheral block □

-general anesthesia a □

-no lower limb surgery in my Hospital □

10. What is your level of knowledge in central blocks?

-no knowledge □

-little knowledge □

-average knowledge□

-very good knowledge □

-expertise□

11. What is your level of knowledge in central blocks with ultrasound guidance?

-no knowledge □

-little knowledge □

-average knowledge□

-very good knowledge □

-expertise□

12. What is your level of knowledge in peripheral blocks with neurostimulation?

-no knowledge □

-little knowledge □

-average knowledge□

-very good knowledge □

-expertise□

13. What is your level of knowledge in peripheral blocks with ultrasound guidance?

-no knowledge □

-little knowledge □

-average knowledge□

-very good knowledge □

-expertise

14. Do you use epidural catheters?
-often
-occasionally
-never
-I do not do epidurals

15. Do you use catheters in peripheral nerve blocks?

-never

-yes, for 24 hours

-yes, for 2–3 days

-I do not perform peripheral blocks

16. What is the main reason for performing regional anesthesia?

-regional anesthesia is safe

-regional anesthesia improves outcome

-regional anesthesia decreases cost of hospitalization

-regional anesthesia decreases the incidence of complications

-regional anesthesia ensures superior postoperative analgesia

-all of the above

17. What is the main obstacle in performing regional anesthesia?

-lack of education

-time-consuming

-patients negative

-surgeons negative

-high percentage of lack of success

-significant percentage of complications

Part II. Regional Anesthesia Course

18. Do you know about the Regional Anesthesia Course?

-yes

-no

19. Have you attended the Regional Anesthesia Course?

-yes

-no

20. What was the main reason for attending the Course?

-to improve my CV

-to improve my knowledge and skills in regional anesthesia

- because of intention to take the European Diploma in Regional Anesthesia (EDRA) exam

-I have not attended the Course

21. How much did the Course contribute to your knowledge about central blocks?

-not at all

-a little

-considerably

-a lot ☐

-I have not attended the Course ☐

22. How much did the Course contribute to your knowledge about central blocks with ultrasound guidance?

-not at all ☐

-a little ☐

- considerably ☐

-a lot ☐

-I have not attended the Course ☐

23. How much did the Course contribute to your knowledge about peripheral blocks with neurostimulation?

-not at all ☐

-a little ☐

-considerably ☐

-a lot ☐

-I have not attended the Course ☐

24. How much did the Course contribute to your knowledge about peripheral blocks with ultrasound guidance?

-not at all ☐

-a little ☐

-considerably ☐

-a lot ☐

-I have not attended the Course ☐

25. How much did the Course change your practice afterwards?

-not at all ☐

-a little ☐

-considerably ☐

-a lot ☐

-I have not attended the Course ☐

26. After the Course and the experience you got from it, have you started performing blocks with ultrasound guidance?

-yes (I was already performing) ☐

-yes, I got more confidence ☐

-no, I still lack the confidence ☐

-no, there is lack of equipment in my Hospital ☐

-no, no suitable operations in my Hospital (i.e., orthopedics) ☐
-I have not attended the Course ☐

27. Did the Course lead to efforts to acquire equipment for regional anesthesia techniques?
-yes ☐
-no ☐
-equipment already available ☐
-I have not attended the Course ☐

28. After your personal experience, would you recommend the attendance of the Course?
-yes ☐
-no ☐
-I have not attended the Course ☐

29. After your personal experience, do you think the Course contributes to regional anesthesia education of Greek anesthetists?

-not at all ☐
-a little ☐
-considerably ☐
-a lot ☐
-I have not attended the Course ☐

30. Please make suggestions about improvement of the Course you attended

-

- I have not attended the Course ☐

31. Can you cite the reason for not having attended the Course?

-because of its cost ☐

-lack of time ☐

-not a priority so far ☐

-I could not reserve a position ☐

-I am not interested in regional anesthesia ☐

-other reasons ☐

-I have attended the Course and I have expressed my opinion above ☐

32. Despite having not attended the Course, do you think that it is of value in Greek anesthesiologists' education in Regional Anesthesia?

-not at all ☐

-a little ☐

-considerably ☐

-a lot ☐

-I have attended the Course and I have expressed my opinion above ☐

33. Are you planning to take the European Diploma in Regional Anesthesia (EDRA) exam?

-yes ☐

-no ☐

-I do not know about this exam ☐

Do you consent to the use of provided anonymized data of this questionnaire for research purposes?

-yes ☐

-no ☐
